# The Accuracy of Different Modalities Used for Preoperative Primary Tumour Localisation in Operated Colorectal Cancer Patients

**DOI:** 10.7759/cureus.36737

**Published:** 2023-03-27

**Authors:** Mahmoud Elnaggar, Ponnuthurai Pratheepan, Baskaran Paramagurunathan, Josie Colemeadow, Basim Hussein, Varvara Bashkirova, Kavya Pillai, Lucy Singh, Mehar Chawla

**Affiliations:** 1 Colorectal Surgery, North Middlesex University Hospital NHS Trust, London, GBR; 2 General Surgery, North Middlesex University Hospital NHS Trust, London, GBR

**Keywords:** primary neoplasm location, colorectal neoplasia, colorectal cancer, ct accuracy, colonoscopy, ct

## Abstract

Aim

Colonoscopy and computed tomography (CT) scans of the abdomen and pelvis are routine pre-operative assessment tools in colorectal cancer (CRC) patients. There have been some discrepancies regarding the location of cancer when seen by colonoscopy versus CT scan.

The purpose of this study was to compare the accuracy of a colonoscopy with a computed tomography (CT) scan of the abdomen and pelvis with contrast, which is done routinely before surgery to localise the exact site of the tumour within the large bowel, whilst comparing both to the operative, gross and histopathology findings of the exact location.

Methods

A retrospective study was carried out on 165 colorectal cancer patients operated on between January 1, 2010, and December 31, 2014, using electronic hospital records that were reviewed anonymously, comparing the location of cancer within the large bowels as was found on colonoscopy and CT scan of the abdomen and pelvis with contrast, comparing both to post-operative histopathology specimen or intra-operative assessment in cases where no resection of the primary tumour was performed.

Results

CT and colonoscopy were both accurate in diagnosing 70.5% of cases that had done both investigations pre-operatively. The best results were obtained when the cancer was located in the caecum as confirmed post-operatively; the combined accuracy rate was 100%. CT was accurate, whilst colonoscopy was not in eight (6.2%) cases (all are rectal or sigmoid cancers), and colonoscopy was accurate and CT was not in 12 cases, 10 of them were rectal and two were ascending colonic. Colonoscopy was not performed in 36 (21%) cases for a variety of reasons, including large bowel obstruction or perforation on presentation. In 32 of these cases, CT scan managed to accurately predict the location of cancer (mostly rectal and caecal), and CT scan was inaccurate in 20.6% of cases (34 out of 165), whilst colonoscopy was inaccurate in 13.9% of cases (18 out of 129).

Conclusion

Colonoscopy is more accurate in localising colorectal cancers than CT scan of the abdomen and pelvis with contrast. CT scan diagnoses regional and distant spread of colorectal cancers such as nodal status, invasion of neighbouring organs and/or peritoneum and the presence of liver metastases, whilst colonoscopy is limited to intraluminal diagnosis but can be both a diagnostic and therapeutic tool, with higher accuracy, in general, in localising colorectal cancers. Both CT scan and colonoscopy were equal in appendicular, caecal, splenic flexure and descending colon cancer localisation accuracy.

## Introduction

Colorectal cancer (CRC) is a global health problem that kills almost 700,000 people each year [1]. CRC is the third most commonly diagnosed cancer worldwide and the second most commonly diagnosed cancer in Europe [1].

Multiple radiographic and endoscopic modalities are used to pre-operatively localise colorectal malignancies, including double-contrast barium enema (DCBE). Barium is used to coat the mucosa, and air is used to distend the colon, with both being inserted transrectally. The DCBE is a fairly safe procedure; however, its use has declined significantly. For large polyps (>10 mm), DCBE has a reported sensitivity of about 50%, and false-positive results might arise due to improper bowel preparation [2,3].

By reconstructing computed tomography (CT) or magnetic resonance imaging (MRI) pictures of the air-distended colon, computed tomographic colonography is a technique that produces two- and three-dimensional endoluminal imaging of the colon. This concept was first described over two decades ago [4,5]. Depending on the study, the diagnostic utility of computed tomographic colonography (CTC) was highly variable. However, as better CTC techniques are developed, it is beginning to match colonoscopy’s sensitivity and specificity for detecting colorectal cancers. In a recent meta-analysis, CTC’s overall sensitivity and specificity were 66.8% and 80.3%, respectively, which were both lower than the outcomes for colonoscopy [6].

CRC screening using colon capsule endoscopy (CCE) was launched in 2006 [7]. Although CCE is more expensive, lacks excision/biopsy capability and necessitates significant intestinal preparation, it may be a better option for the patient than colonoscopy [8,9].

Colorectal cancer screening with a colonoscopy is the gold standard, and it has a reported sensitivity of detection ranging from 85% to 96% of malignancies [10-13]. It is also one of the main investigations used in the localisation of colorectal cancers with or without India ink as an adjunct [14-16]. Its sensitivity and specificity in localising colorectal cancer have been well documented in the literature [17-19].

Because synchronous adenomas and carcinomas are common in patients with colorectal cancer, a careful colonoscopy is very important. Meanwhile, occlusive colorectal cancer makes standard colonoscopy impractical in roughly 6%-26% of cases [20-23].

CT scan of the abdomen and pelvis is a routine scan (combined with CT chest) done not only for pre-operative assessment of the location of cancer within the large bowel but also to determine the nodal status, invasion of surrounding structures and metastasis to other organs and for tumour staging. The role of conventional CT in assessing patients with colorectal tumours is well established. CT can be used to examine individuals who are suspected of having extensive disease, to determine whether or not they will benefit from pre-operative radiation, construct radiation ports and detect problems connected to the tumour [24].

The development of laparoscopic and laparoscopic-assisted colectomy has increased the need for the accurate pre-operative localisation of colonic lesions [17,18]. This is also true for screen-detected lesions where a palpable mass might not be present following endoscopic polypectomy [10].

The most common method for staging colon cancer is a CT scan of the abdomen and chest [25]. It should preferably be performed as a multiphase contrast-enhanced CT with an arterial phase for the liver and a venous phase for the chest and abdomen [26]. It permits the detection of ascites, severe carcinomatosis and invasion into surrounding organs, as well as bulky lymphadenopathy. In the majority of instances, liver metastases greater than 1 cm in diameter and lung metastases can be detected with up to 95% accuracy [27]. Normal imaging characteristics can be employed with reasonable accuracy to identify lesions for tumour (T) and node (N) staging in colon cancer, with accuracies ranging from 67% to 69%. The T stage and nodal stage subclassifications, with the exception of T4 tumours, have no bearing on surgical therapy and are hence unnecessary in the pre-operative workup [28-30]. In people suspected of having advanced CRC, CT scans are now considered standard pre-operative evaluation [31-33]. According to Mauchley et al. [31], 16% of patients benefit from routine pre-operative CT because it provides valuable information as to treatment planning and is cost-effective.

## Materials and methods

This is a retrospective study of colorectal cancer patients operated on between January 1, 2010, and December 31, 2014, using electronic hospital records that were reviewed anonymously, comparing the location of cancer within the large bowels as was found in post-operative histopathology specimen or by intra-operative assessment (in cases where no resection of the primary tumour was performed) by the two modalities used for the diagnosis and localisation of cancer pre-operatively: CT scan of the abdomen and pelvis, and colonoscopy.

**Figure 1:**
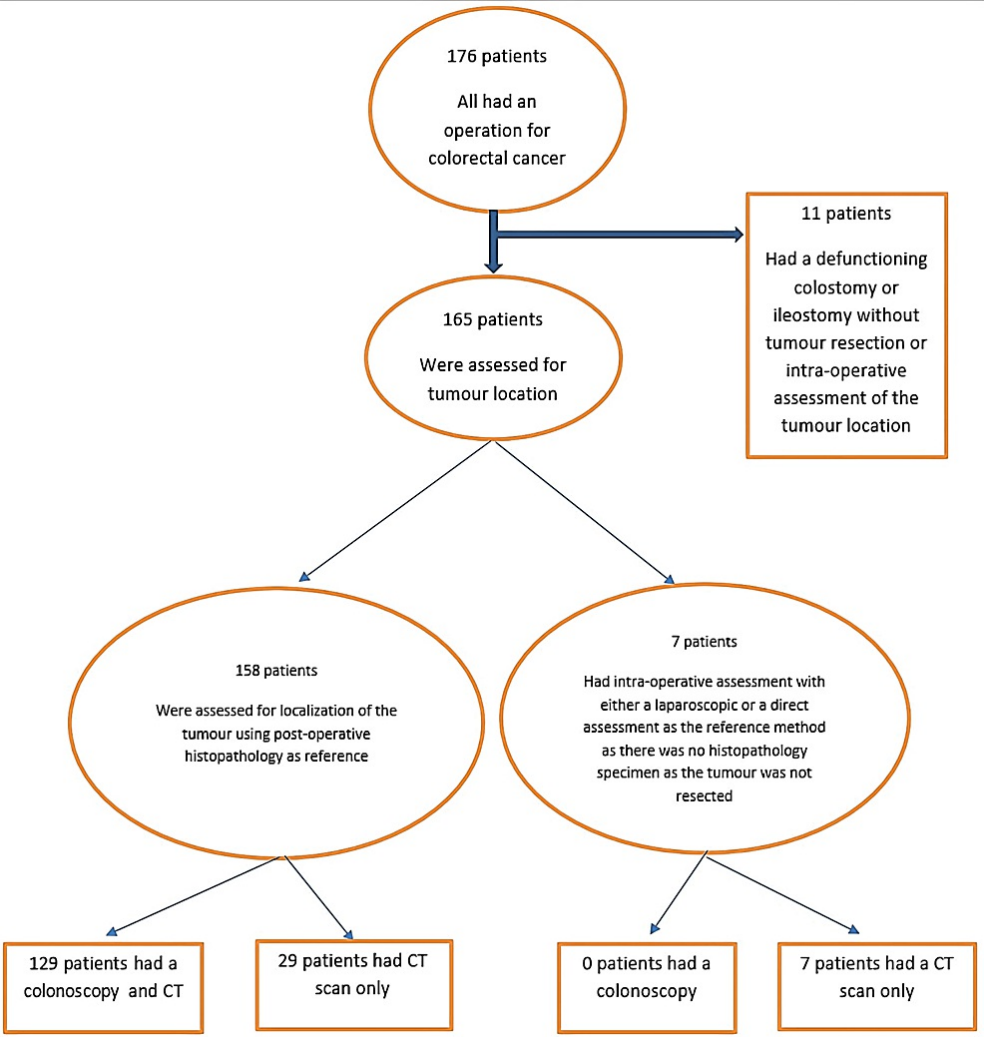
Exclusion criteria and distribution of patients according to the assessment method CT: computed tomography

There were 176 patients with an operation for colorectal cancer. In 11 patients, the tumour was assessed as inoperable intra-operatively, and only a stoma was created without tumour resection, with no intraoperative assessment of the location of the tumour. Thus, the total number of patients that the study included in the end after the exclusion of these 11 patients was 165 patients. These patients were assessed for tumour location on post-operative histopathology (158 patients) or intra-operatively (seven patients, where the tumour was inspected and localised either laparoscopically or during an open operation, with no histopathology as the tumour was not resectable) (Figure 1).

Comparisons were made on the accuracy of the localisation of the primary tumour using the pre-operative localisation modalities CT scan of the abdomen and pelvis in 165 cases and colonoscopy in 129 cases. Colonoscopy was not performed in 36 cases for a variety of reasons, including large bowel obstruction or perforation on presentation. In these cases, pre-operative CT scan results were compared to the intra-/post-operative results.

## Results

A total of 165 patients were assessed for localisation of the tumour using post-operative histopathology as a reference. This was done in 158 patients; the other seven patients had an intra-operative assessment, either laparoscopic or direct assessment as the reference method, as there was no histopathology specimen, as the tumour was not resected. Most of the cancers were rectal (33.9%), sigmoid (24.2%) and caecal (21.2%) (Table 1).

**Table 1:** Location of the tumour as decided post-operatively

Site	Number	%
Appendix	3	1.80%
Caecum	35	21.20%
Ascending colon	10	6%
Hepatic flexure	7	4%
Transverse colon	6	3.60%
Splenic flexure	4	2.40%
Descending colon	4	2.40%
Sigmoid colon	40	24.20%
Rectum	56	33.90%
Total	165	100%

There were no cases recorded without a pre-operative assessment CT scan, whether CT scan of the abdomen and pelvis or with added chest CT scan (CT cap), but there were 36 cases without pre-operative colonoscopy. There were eight cases where the CT scan could not detect the cancer, whilst there were no cases when a colonoscopy was done that the tumour was not detectable. CT and colonoscopy were both accurate in 70.5% of the cases when both modalities were done; the highest score for the accuracy of both modalities combined was in caecum, appendix and descending colon cancers (100%) (Table 2).

**Table 2:** Tumour detection accuracy of CT and colonoscopy according to the location of colorectal cancer

Post-operative cancer location when both CT scan and colonoscopy were done pre-operatively	Number	CT correct, colonoscopy correct	CT correct, colonoscopy incorrect	CT incorrect, colonoscopy correct	CT incorrect, colonoscopy incorrect	CT not detecting the cancer, colonoscopy correct	Colonoscopy not detecting the cancer
Appendix	3	3 (100%)	0 (0%)	0 (0%)	0 (0%)	0 (0%)	0 (0%)
Caecum	21	21 (100%)	0 (0%)	0 (0%)	0 (0%)	0 (0%)	0 (0%)
Ascending colon	8	4 (50%)	0 (0%)	2 (25%)	2 (25%)	0 (0%)	0 (0%)
Hepatic flexure	7	3 (42.8%)	0 (0%)	0 (0%)	4 (57.2%)	0 (0%)	0 (0%)
Transverse colon	6	2 (33.3%)	0 (0%)	0 (0%)	4 (57.2%)	0 (0%)	0 (0%)
Splenic flexure	4	4 (100%)	0 (0%)	0 (0%)	0 (0%)	0 (0%)	0 (0%)
Descending colon	4	4 (100%)	0 (0%)	0 (0%)	0 (0%)	0 (0%)	0 (0%)
Sigmoid colon	34	24 (66.6%)	6 (17.6%)	0 (0%)	0 (0%)	4 (11.7%)	0 (0%)
Rectum	42	26 (61.9%)	2 (4.7%)	10 (23.8%)	0 (0%)	4 (9.5%)	0 (0%)
Total	129	91 (70.5%)	8 (6.2%)	12 (9.3%)	10 (7.7%)	8 (6.2%)	0 (0%)

**Figure 2:**
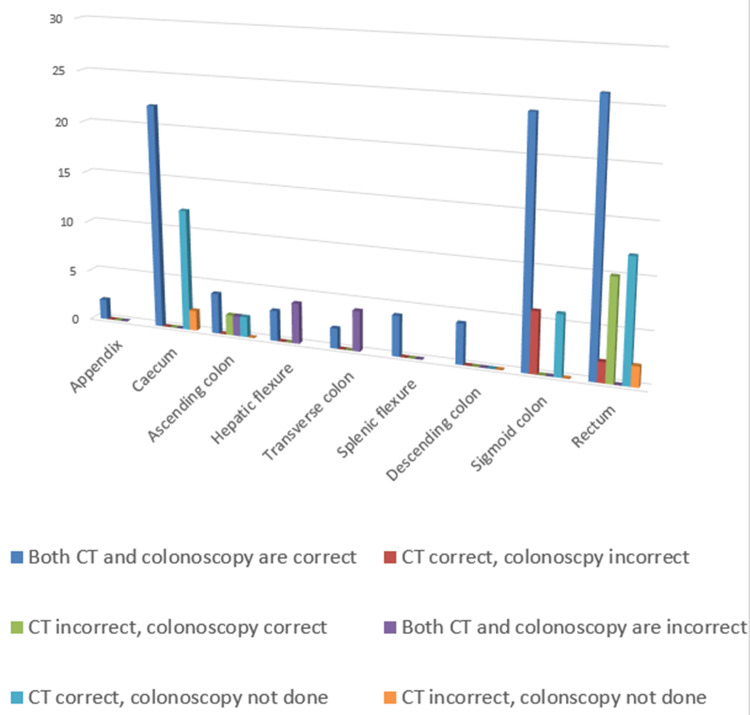
Accuracy of CT scan and colonoscopy according to postoperative findings CT: computed tomography

Both investigations were inaccurate in detecting the actual tumour location in 10 (7.7%) cases, when the tumour was located in the transverse colon, hepatic flexure (four cases each) and ascending colon (two cases). CT scan was accurate, whilst colonoscopy was not in eight (6.2%) cases (all are rectal or sigmoid cancers). The opposite happened in 12 (9.3%) cases; 10 of them were rectal, whilst two were ascending colonic. CT and colonoscopy both had false results in 10 cases out of 129 cases where both investigations were done, and it is worth noting that all of these cancers were on the right side of the colon (Table 2, Figure 2).

As mentioned earlier, colonoscopy was not performed in 36 (21.8%) cases for a variety of reasons, including large bowel obstruction or perforation on presentation; in 32 (88.9%) of these cases, CT scan managed to accurately predict the location of cancer (mostly rectal and caecal) (Table 3).

**Table 3:** Accuracy of CT when done alone preoperatively according to the location of cancer post-operatively CT: computed tomography

	Only a CT was done; colonoscopy was not done (%)	Only a CT was done and is correct (%)	Only a CT was done and is incorrect (%)
Appendix	0 (0%)	0 (0%)	0 (0%)
Caecum	14 (40% of total caecal cancers diagnosed)	12 (85.7%)	2 (15.3%)
Ascending colon	2 (20% of total ascending colon cancers diagnosed)	2 (100%)	0 (0%)
Hepatic flexure	0 (0%)	0 (0%)	0 (0%)
Transverse colon	0 (0%)	0 (0%)	0 (0%)
Splenic flexure	0 (0%)	0 (0%)	0 (0%)
Descending colon	0 (0%)	0 (0%)	0 (0%)
Sigmoid colon	6 (15% of total sigmoid cancers diagnosed)	6 (100%)	0 (0%)
Rectum	14 (25% of total rectal cancers diagnosed)	12 (85.7%)	2 (15.3%)

There was a 100% detection rate by both CT and colonoscopy in appendicular, caecal, splenic flexure and descending colon cancers when both modalities were done pre-operatively (Table 2).

**Figure 3:**
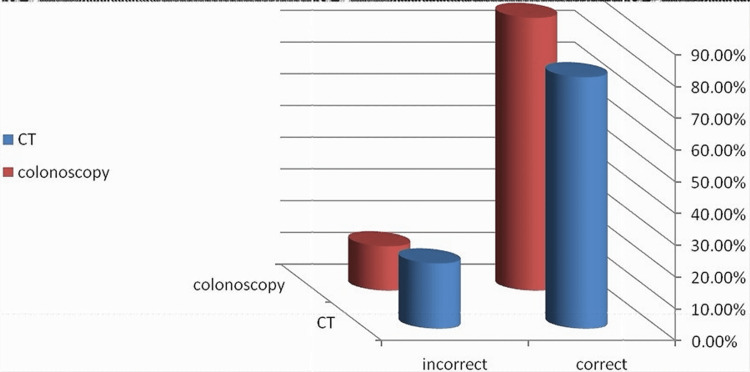
CT versus colonoscopy: overall accuracy in predicting cancer location pre-operatively CT: computed tomography

The cumulative CT scan accuracy for the 165 cases, when a CT scan was done pre-operatively, showed that the CT scan was inaccurate in 20.6% (34 out of 165) of cases, whilst colonoscopy was inaccurate in 14% (18 out of 129) of cases (Figure 3).

The operative decision was changed intra-operatively in 16 patients, or 9.7% of the study patients, based on inaccuracy in the pre-operative investigations. This was most evident in rectal cancers thought to be highly sigmoidal according to pre-operative localisation (Table 4).

**Table 4:** Changes in planned surgical management due to altered intra-operative lesion location TME: total mesorectal excision

Planned	Actual	Reason	Number of cases
Extended right hemicolectomy	Right hemicolectomy	Lesion in the ascending colon rather than in the transverse colon	2
Right hemicolectomy	Extended right hemicolectomy	Lesion in the transverse colon rather than in the ascending colon	4
Anterior resection	Full TME	Sigmoid to lower rectum	7
Left hemicolectomy	Anterior resection	Descending actually distal sigmoid	3

## Discussion

Patients with synchronous adenomas and adenocarcinomas are present in 14%-48% and 2%-9% of CRC cases, respectively; in these patients, a complete large bowel examination is crucial for treatment planning [34-42]. The gold standard for finding colon cancer is a colonoscopy, but tumour obstruction, which is common in distal malignancies, may render it ineffective [23,43].

To avoid needless adjuvant therapy and select the best surgical option, such as segmental sigmoid resection, low anterior resection or abdominoperineal resection, accurate tumour localisation is particularly crucial for rectal carcinomas [10]. The distance between the bottom edge of the tumour and the external sphincter and levator ani muscle should be measured if the anal sphincter is to be preserved. This was highlighted in the current study, as shown in Table 4, where pre-operative planning of the surgery needed to be changed intra-operatively due to the discovery of the tumour in a different location. This happens mainly in low rectal cancers thought to be sigmoid, which needed full total mesorectal excision (TME) with stomas needed in some cases for the protection of low anastomosis, which has significant implications on patients who do not expect a stoma as an outcome of their operations. Another prospective multi-centre observational study by Johnstone et al. [44] reported that colonoscopic lesion localisation was incorrect in 19% of patients and occurred throughout the colon, with a change in on-table surgical therapy in 6.3% of cases.

As shown in Figure 3, the current study showed an overall colonic accuracy of 86% with an erroneous localisation rate of 14%, while the research by Stanciu et al. [45] showed that 161 patients (89 males and 72 females aged 61.3±12.8 years) had surgeries for colon cancer following its discovery by colonoscopy during this study period, with an overall colonic accuracy of 86% and an erroneous localisation rate of just 14%. Erroneous colonoscopic localisation of tumours was found in 22 (13.66%) patients. They concluded that colonoscopy is an accurate and reliable way of identifying colon cancer, albeit other procedures (such as endoscopic tattooing) should be used at least for small lesions.

Saleh et al. [46] studied 298 patients, 118 (39.6%) of whom had a pre-operative re-endoscopy performed by the operating surgeon or a colleague. At the initial endoscopy, 19 individuals had the wrong tumour location, resulting in a 6.4% mistake rate (95% confidence interval (CI): 3.88-9.78). On re-endoscopy, there were two localisation mistakes, totalling 1.69% (95% CI: 0.21-6.00). Re-endoscopy was found to be protective against localisation errors (P=0.05), with 10 of the 12 errors committed during the initial endoscopy being corrected. The sensitivity of re-endoscopy as a diagnostic tool to detect errors was 83%, with a corresponding specificity of 100%. The overall accuracy of re-endoscopy in preventing endoscopic localisation errors was 92% (95% CI: 81-100). They concluded that in colorectal tumour pre-operative endoscopic examination, there is a minor but significant rate of localisation inaccuracy. Re-endoscopy appears to be safe and may aid with pre-operative planning, and identifying and correcting these problems. More research is needed to improve localisation and determine whether patients will benefit from repeat endoscopy.

A study by Cho et al. [17] showed similar accuracy of pre-operative colonoscopy to the current study results. Between April 2000 and March 2006, 310 patients who underwent laparoscopy-assisted colectomy were studied. They looked at whether the pre-operatively estimated tumour locations were congruent with the actual tumour locations after the operation. In 23 cases, colonoscopy failed to accurately locate tumours (11.3%). A barium enema was performed on 104 (33.5%) patients; five (4.8%) tumours were not visible, and three tumours were incorrectly placed. Another group of 94 (30.3%) patients had colonography done with computed tomography, which detected 91 of the 94 anomalies (96.8%). Finally, 96 (31%) patients underwent endoscopic tattooing; two (2.1%) patients had tattoos that were not visible laparoscopically and required intra-operative colonoscopy to find their tumours during removal. Colonoscopy (180/203, 88.7%), barium enema (97/104, 93.3%), CT colonography (89/94, 94.7%), endoscopic tattooing (94/96, 97.9%) and intra-operative colonoscopy (4/4, 100%) were the most accurate methods for tumour localisation.

The Accurate Lesion Localisation at Colonoscopy (ALLaC) study involved eight UK hospitals [47]. In this study, colonoscopy was found to wrongly localise colorectal lesions in 18% of instances, resulting in 5.2% of cases requiring surgical care in the operating room. Because the data was collected after the colonoscopy, rather than during it, several colonoscopists would have been blinded. When the locations of colonoscopy and CT were combined, the percentage of proper lesion localisation climbed to 87.1%. However, this study found that CT imaging fails to detect lesions in more than a quarter of cases, especially among the screening group. The National Health Service Bowel Cancer Screening Program (NHSBCSP) is predicted to continue to detect earlier and smaller lesions.

Colonoscopy’s importance in optimal pre-operative surgical planning is likely to grow, and we can see the similarity in the outcome regarding colonoscopy with the current study; alteration in surgical management occurred in a higher percentage in the current study (9.7%), CT did not visualise cancer in 6.2% of cases, which is a much lower incidence of complete CT failure to identify cancer, and colonoscopy was able to do so in all of these patients.

Bayrak et al. [48] looked at 156 patients to see how accurate tumours were located generally. The rectum, sigmoid colon and rectosigmoid junction (RSJ) were studied using colonoscopy, computed tomography, magnetic resonance imaging (MRI) and fluoro-deoxy-glucose/positron emission tomography-computed tomography (FDG/PET-CT) to determine the location of distal colorectal cancers. Colonoscopy, CT, MRI and FDG/PET-CT showed 74%, 67%, 75% and 74% accuracy rates, respectively. Colonoscopy was less sensitive for rectosigmoid tumours (33%), while CT was less sensitive for rectal tumours (26%). In comparison to the current study, CT scan failed to accurately localise 16 out of 56 (38.2%) rectal cancers, whereas colonoscopy had better results for rectosigmoid cancers, with failure to accurately localise only one out of every 10 rectal cancers.

Solon et al. [49] conducted another study, where 73 (73%) of 101 individuals had adenoma or cancer, with adenocarcinoma being the ultimate diagnosis in 59 (58%) of them. Both CT and colonoscopy failed to accurately localise 29% of tumours before surgery. Colonoscopy alone was only 37.5% accurate in the transverse colon, but it increased to 62.5% when CT scan information was added. It was found that pre-operative localisation of right-sided colon malignancies using colonoscopy plus CT scanning was incorrect in at least 29% of instances. Inaccurate transverse colon tumour localisation can lead to insufficient lymphadenectomy and poor cancer result. Although a pre-operative CT scan of the abdomen enhances accuracy, endoscopic tattoo localisation should be used regularly, especially in patients undergoing laparoscopic resection. The findings of this study were that the overall failure of both modalities to localise right-sided colon cancers was 12 out of 58 caecal, ascending colonic, hepatic flexure and transverse colon (20.6%).

## Conclusions

Colonoscopy is more accurate in localising colorectal cancers than CT scan of the abdomen and pelvis with contrast. CT scan has an accuracy of 79.3%, while colonoscopy has an accuracy of 86% in localising colorectal cancers, with the highest detection rates of 100% by both CT scan and colonoscopy in appendicular, caecal, splenic flexure and descending colon cancers. Both tools are still important pre-operative checks with the added value of CT scan diagnosing regional and distant spread of colorectal cancers such as nodal status, invasion of neighbouring organs and/or peritoneum and the presence of liver metastases, while colonoscopy is limited to intraluminal diagnosis. However, colonoscopy has the therapeutic option of inserting a stent into a tumour, but the CT scan lacks such interventional options.

It is particularly important to accurately localise colorectal cancers preoperatively, especially if laparoscopic surgery is planned. The use of colonoscopic tattooing is important due to the absence of tactile localisation. The inability to accurately localise the tumour preoperatively may lead to an intraoperative change in the surgical decision.
